# Acupuncture for Histamine-Induced Itch: Association With Increased Parasympathetic Tone and Connectivity of Putamen-Midcingulate Cortex

**DOI:** 10.3389/fnins.2019.00215

**Published:** 2019-03-12

**Authors:** Seorim Min, Koh-Woon Kim, Won-Mo Jung, Min-Jung Lee, Yu-Kang Kim, Younbyoung Chae, Hyangsook Lee, Hi-Joon Park

**Affiliations:** ^1^Department of Science in Korean Medicine, Graduate School, College of Korean Medicine, Kyung Hee University, Seoul, South Korea; ^2^Acupuncture and Meridian Science Research Center, Kyung Hee University, Seoul, South Korea; ^3^Department of Korean Rehabilitation Medicine, College of Korean Medicine, Kyung Hee University, Seoul, South Korea; ^4^East-West Medical Research Institute, Kyung Hee University, Seoul, South Korea

**Keywords:** acupuncture, histamine, itch, heart rate variability, functional magnetic resonance imaging, putamen, midcingulate cortex

## Abstract

Previous studies have suggested that acupuncture is effective for ameliorating itch intensity. However, factors associated with the antipruritic effects of acupuncture have yet to be clarified. In a randomized, sham-controlled, crossover trial, we investigated the antipruritic effects of acupuncture against histamine-induced itch in healthy volunteers. Autonomic changes using heart rate variability (HRV) and brain connectivity using functional magnetic resonance imaging (fMRI) were also assessed to identify physiological factors associated with the acupuncture response. Acupuncture significantly reduced itch intensity and skin blood perfusion as assessed by laser Doppler perfusion imaging compared to sham control, indicating the antipruritic effects of acupuncture. In responder and non-responder analysis, the power of normalized high frequency (HF norm) was significantly higher, while the power of normalized low frequency (LF norm) and LF/HF ratio were significantly lower in responders compared to non-responders, suggesting the acupuncture response involved parasympathetic activation. In fMRI analysis, the putamen and the posterior part of the midcingulate cortex (pMCC) were positively connected to itch and negatively correlated with itch intensity in responders. These results suggest that parasympathetic activity and functional connectivity of the putamen and pMCC could be associated with antipruritic response to acupuncture.

## Introduction

The sensation of itch, defined as “an unpleasant sensation associated with the desire to scratch,” is the most prevalent subjective symptom of inflammatory skin diseases and cause of suffering in many dermatologic and some allergic conditions (Charlesworth and Beltrani, 2002). Itching leads to scratching, consequently interfering with skin barrier function. With the loss of cutaneous integrity and softening of the skin surface, a vicious cycle of skin damage and inflammation occur. This cycle induces a state of complex dermatitis, creates more itching, resulting in a scratch-itch cycle, and leads to serious quality-of-life problem. Patients with atopic dermatitis (AD) suffer from severe itch, most notably hampering their quality of sleep (Arck and Paus, 2006; Bender et al., 2008).

The autonomic nervous system (ANS) is the primary mechanism for the response to external stimuli. The response results in a fight-or-flight system from the sympathetic nervous system (SNS) or freeze-or-dissociate response from parasympathetic nervous system (PNS). Some studies suggest that the ANS functions in the itch mechanism (Stander and Steinhoff, 2002; Chida et al., 2007; Cicek et al., 2008). A previous study (Kirchner et al., 2002) found that the vagus nerve, which is the main parasympathetic outflow, controls histamine-induced itch, probably by a central mechanism. The brain circuitry involved in the itch sensation is an active topic of study of the peripheral and central sensitization for itch (Desbordes et al., 2015). Itch is usually perceived when inflammatory mediators activate a specific subset of peripheral sensory nerve endings, an activation that is transmitted to the brain via the spinothalamic tract (Andrew and Craig, 2001; Davidson et al., 2007; Napadow et al., 2014).

Many studies have focused on itch itself, but appropriate treatments are far from satisfactory. A systematic review (Yu et al., 2015) suggested that acupuncture is effective for ameliorating itch intensity in itch-related diseases. Topical antihistamines have limited use for acute itch only and have relatively high costs and risks for allergic contact dermatitis sensitization (Callahan and Lio, 2012). A previous study showed that acupuncture has better antipruritic effects than third generation antihistamines (Napadow et al., 2014). Another study comparing acupuncture and second generation antihistamine found that acupuncture demonstrated a greater effect during peak itch intensity, while antihistamine had a stronger effect during lower itch intensity suggesting different mechanisms of action (Pfab et al., 2012a). Acupuncture for experimentally induced itch in healthy participants (Belgrade et al., 1984; Lundeberg et al., 1987; Pfab et al., 2005) and patients with AD (Pfab et al., 2010, 2011, 2012a; Napadow et al., 2014) has a significant antipruritic effect compared to placebo. Possible mechanisms for itch reduction by acupuncture in patients with AD include reduction of *in vitro* allergen-induced basophil activation and modulation of neurotransmitters, peripheral hormone levels, and brain areas that are involved in itch processing (Pfab et al., 2013). Furthermore, acupuncture may reduce itch by creating an inhibitory input called gate-control theory through the response of the sensory nerve with a one-to-one correspondence between dermatomes and spinal segments (Bear et al., 2016). However, factors related to the antipruritic effects of acupuncture remain unclear.

Acupuncture stimulation influences organs and functions including ANS activity (Kawakita et al., 2006) and brain processing (Dhond et al., 2007a,b). Several studies suggest that acupuncture influences ANS functions such as blood pressure, skin conductance, skin temperature, heart rate, and heart rate variability (HRV) (Malliani et al., 1991; Lee et al., 2010; Chung et al., 2014). Studies found that press needle stimulation induces alterations in vagal function (Noda et al., 2015) and acupuncture stimulation at HT7 affects cardiac autonomic neural regulation in healthy individuals, mainly via the PNS (Huang et al., 2015). Acupuncture activates the PNS and default mode network connectivity (Dhond et al., 2008). It induces changes in heart rate and brain stem structures (Beissner et al., 2012).

Using functional magnetic resonance imaging (fMRI), we investigated changes in cerebral perfusion to identify the critical brain areas mediating the antipruritic effect of acupuncture. The fMRI method for imaging brain functions is increasingly used to investigate dynamic brain patterns with acupuncture stimulation (Malliani et al., 1991; Park et al., 2005; Dhond et al., 2007b; Colombo et al., 2015). In clinics, acupuncture treatment typically involves a phase of needle retention following acupuncture insertion and manipulation. Thus, functional connectivity indicating characteristics of acupuncture is better suited for a block design of fMRI analysis. Functional connectivity analysis explores the relationship between neuronal activation patterns that functionally linked, but anatomically separated brain areas (Colombo et al., 2015). The striatum, which consists of the putamen and the caudate, is critical for striato-thalamo-cortical circuits for itch. An fMRI study (Napadow et al., 2014) found itch reduction following acupuncture is associated with reduced activation of the putamen response. Thus, our study chose the putamen as a seed ROI.

Since individually differing responses to acupuncture have been reported and itch is a subjective symptom, an individualistic approach is needed (Park et al., 2005; Chae et al., 2006). Our individualistic approach classified participants into responders and non-responders (Scholz and Woolf, 2002; Woodcock et al., 2007; Schork, 2015). Analysis of the differences between responders and non-responders strengthens therapeutic effects by managing response-related factors (Kim et al., 2007).

The aim of this study was to test the antipruritic effects of acupuncture on experimentally induced itch. Subsequently, we investigated the factors that influenced these effects via ANS response and functional connectivity of the brain by analyzing responder and non-responder groups.

## Materials and Methods

The study was conducted in two separate steps. *Study 1* was followed by *Study 2*.

### Participants

For *Study 1*, 20 healthy participants between 18 and 50 years of age were recruited using an advertisement. Participants with no present history of asthma and AD were screened by telephone. *Study 2* had 15 participants who met the schedule for fMRI scans and retested for MRI compatibility. All participants refrained from alcohol or caffeine for 12 h before experiments. Participants with skin problems on the stimulated site were excluded. Participants had to stop antihistamine medications at least 3 days before the study to avoid potential influence on normal itch perception or flare and wheal response to SPT, nevertheless, in case of no itch response, the applicable data would be excluded for analysis. Detailed explanations of experimental procedures such as the two conditions and four visits were given. This study was carried out in accordance with the recommendations of the guideline for clinical researches, Kyung Hee University Ethics Committee with written informed consent from all subjects. All subjects gave written informed consent in accordance with the Declaration of Helsinki. The protocol was approved by the Kyung Hee University Ethics Committee (KHSIRB-15-056).

### Experimental Design

The study design was a randomized, crossover trial in which each participant served as his or her own control. Participants received acupuncture and placebo stimulation in random order. Session orders were determined with a random number table generated by Excel (Microsoft, Redmond, WA, United States). Each experiment was separated by 7 days and occurred at the same time of the day. Tests were carried out in winter, in January to March 2016, to minimize the influence of environmental allergens.

**FIGURE 1:**
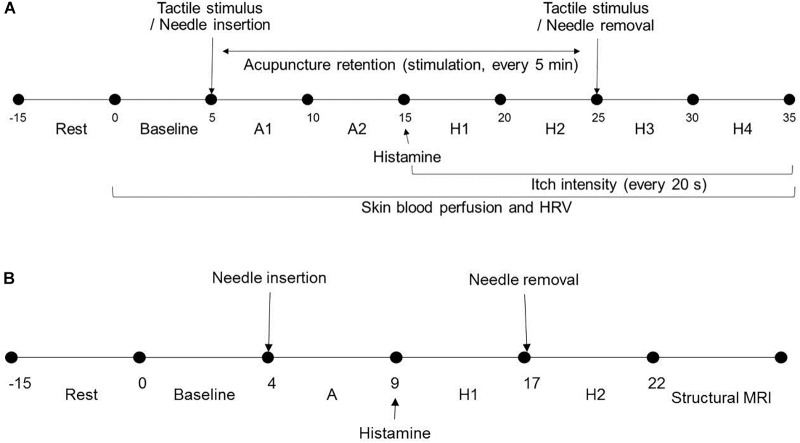
Study design and experimental procedures: **(A)** In *Study 1*, after resting, baseline skin blood perfusion and HRV were obtained for 5 min and intervention was subsequently performed on the non-dominant side. Skin blood perfusion and HRV were obtained (A1 and A2 phase) for 10 min. After itch induction, HRV, skin blood perfusion, and itch intensity were measured for 10 min (H1 and H2 phase). The intervention was removed and skin blood perfusion, HRV, and itch intensity were obtained (H3 and H4 phase). **(B)** In *Study 2*, after 15 min resting, participants underwent three fMRI scanning sessions. Scans for each session were for 4-min baseline resting state, intervention (A, 5 min), and itch resting state (H1, 0–4; H2, 4–8 min). HRV, heart rate variability; s, seconds; min, minutes; fMRI, functional magnetic resonance imaging.

*Study 1* was in a light-conditioned and quiet room. Room temperature was controlled at 23 ± 1°C (mean ± SD). Demographic data including gender, age, height, and weight and psychological factors such as credibility, expectation, and fear of acupuncture and perception of bodily sensation were collected from all participants. After 15 min of resting, participants were seated on a chair throughout and remained comfortable in that position. Baseline skin blood perfusion and HRV measurements were obtained for 5 min and interventions were subsequently performed on the non-dominant side. Skin blood perfusion and HRV were obtained (A1 and A2 phase) for 10 min. After itch induction, HRV, skin blood perfusion and itch intensity were measured for 10 min (H1 and H2 phase). The intervention was removed and skin blood perfusion, HRV, and itch intensity were obtained (H3 and H4 phase) (Figure 1A).

**FIGURE 2:**
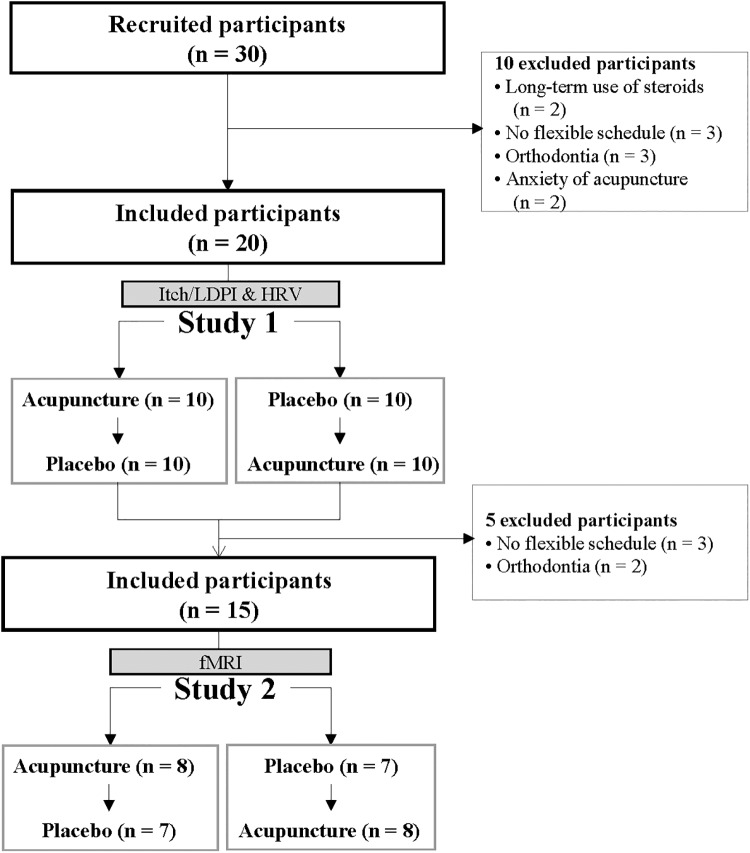
Study flow: participant flow through *Study 1* and *Study 2*.

For *Study 2*, room temperature was controlled at 11 ± 1°C (mean ± SD). As participants lay in the magnet, they viewed a screen using prism glasses to respond to situations by pressing a button. After 15 min of resting, participants underwent three fMRI scanning sessions. Each session included a 4-min baseline resting state scan, intervention (5 min), and itch resting state scan (0–4, 4–8 min) (Figure 1B). The order of acupoints and rest periods within each session were counterbalanced and randomized across participants. Itch was induced as in *Study 1*. The study flow is depicted in Figure 2.

### Acupuncture and Placebo Control

For *Study 1*, manual acupuncture stimulation was used on the arm and leg of the participant’s non-dominant side at LI11 (on the elbow at the midpoint of the end of the transverse cubital crease and lateral epicondyle of the humerus when the elbow is flexed); SP10 (on the medial aspect of the thigh, 6.6 cm above the medio-superior border of the patella, on the bulge of the medial portion of muscle quadriceps femoris when the knee is flexed); PC6 (on the palmar aspect of the forearm, 6.6 cm proximal to the middle point of the carpal fold, on the line connecting PC3 and PC7, between the tendons of muscle palmaris longus and muscle flexor carpi radialis); HT7 (on the wrist, at the ulnar end of the transverse crease, in the depression on the radial side of the tendon of the ulnar flexor muscle); and ST36 (on the anterior aspect of the lower leg, 9.9 cm below ST35, middle finger from the anterior crest of the tibia). Acupoints based on a standard acupuncture textbook, previous study, and clinical experience, are important for treating cutaneous itch. LI11 and SP10 are used frequently to treat itch in clinical and experimental studies (Pfab et al., 2005, 2010, 2011, 2012a). To induce activation of the PNS, HT7 (Huang et al., 2015) and PC6 (Huang et al., 2005; Li et al., 2005) were chosen. ST36 is the most frequently used acupoint for immune regulation and anti-inflammatory effects, and reducing SNS (Michikami et al., 2006). For *Study 2*, due to fMRI system structural problems, LI11 was excluded.

**FIGURE 3:**
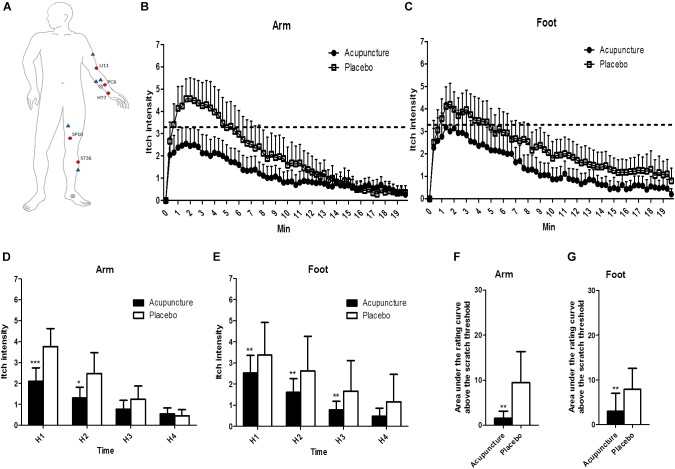
The location of treatments and changes of itch intensity: **(A)** The locations of the acupoints in the acupuncture and the control points and the histamine-stimulated site. Red circles indicate acupoints in the acupuncture group, and blue triangles indicate placebo points. X marks indicate the location of histamine injection. **(B,C)** Itch intensity over time after histamine stimulus in the arm and the foot. The dotted line indicates scratch threshold (3.3/10). Itch intensities for entire measurements were significantly lower in acupuncture compared to the placebo condition for arm (*p* < 0.001) and foot (*p* < 0.001) in two-way ANOVA. **(D,E)** Area under the rating curve above the scratch threshold in the arm and the foot. **(F,G)** Mean of itch intensity by 5-min intervals after histamine stimulus in the arm and the foot. Values are mean (95% CI). *P*-values show results of paired sample *t*-test or Wilcoxon signed rank test for comparison of itch intensity between two conditions at each time point. ^∗^*p* < 0.05, ^∗∗^*p* < 0.01, ^∗∗∗^*p* < 0.001. Relative to histamine stimulus, H1 is 0–5 min, H2 is 5–10 min, H3 is 10–15 min, and H4 is 15–20 min.

For *Study 1*, sterile stainless steel needles (0.20 mm in diameter, 30 mm in length; Haeng Lim Seo Won, South Korea) were inserted to depth (LI11, 20 mm; SP10, 15 mm; PC6, 15 mm; HT7, 6 mm; ST36, 20 mm) controlled by an empty guide tube and manipulated for 9 s repeated every 5 min over 20 min (Figure 3A). For *Study 2*, non-magnetic titanium sterile acupuncture needles (0.20 mm diameter, 40 mm length; DongBang Acupuncture Inc., Boryeong, South Korea) were used. Manipulation techniques were performed as tonifying and reducing, which involved bidirectional rotation (1 Hz). A previous study (Takakura et al., 1995; Min et al., 2015) showed repeated manipulation improves acupuncture effects and traditionally, manual acupuncture uses manipulation to reinforce effects (Kim et al., 2000). The same doctor of Korean medicine with a certified license performed all acupuncture treatments. Prior to baseline, skin was cleaned with alcohol at the acupoint and histamine stimulus locations.

As a placebo control, tactile stimulation was performed by gentle tapping with a size 5.46 von Frey filament (Touch-Test Sensory Evaluator Instructions, North Coast Medical, Inc., CA, United States) every 5 min over 20 min for *Study 1* and over 5 min for *Study 2*. Placebo acupoints were on the arm and leg using the same meridian system, but not acknowledged as acupoints by textbooks. The non-acupoint for LI11 was on the lateral side of the arm at the midpoint of the line connecting LI11 and LI15. The non-acupoint for SP10 was on the medial aspect of the thigh at the midpoint of the line connecting SP10 and SP12. The non-acupoint for PC6 was on the palmar aspect of the forearm at the midpoint of the line connecting PC3 and PC7. The non-acupoint for HT7 was on the palmar aspect of the forearm at the midpoint of the line connecting HT3 and HT7. The non-acupoint for ST36 was on the anterior aspect of the lower leg in the center of the belly of the muscle tibialis anterior. Placebo procedures were carried out as acupuncture procedures. The von Frey filament and acupuncture materials were prepared in advance and placed on a tray and covered so materials could not be seen by participants. Locations of acupoints and non-acupoints were blinded by self-produced blinding box. More detailed procedures according to the Standard for Reporting Interventions in Clinical Trials of Acupuncture (STRICTA) are attached in Supplementary Appendix 1.

### Itch Induction

After acupuncture procedures, 1% histamine (10 mg/ml histamine dihydrochloride, Lofarma, Milan, Italy) was applied to induce itch on the volar aspect of the forearm and medial aspect of the foot of the participant’s non-dominant side using SPT. A single drop of histamine in aqueous solution was applied to the skin, followed by puncture with a lancet for SPT as in routine allergy diagnosis. The technique was performed by the same investigator.

### Itch Intensity

For *Study 1*, itch intensity was rated at 20-s intervals for 20 min after SPT. Participants were seated with the left arm resting on the cushion of a chair arm and the other hand selecting a number from 0 (no itch) to 10 (worst imaginable itch). One-third of the scale (3.3/10) was the intervention point “scratch threshold,” above which the individual felt a strong desire to scratch, which was not allowed. Area under the rating curve above the scratch threshold was calculated as itch intensity multiplied by time (in min). For *Study 2*, participants could view a screen in the scanner through prism glasses in the supine position. Itch intensity was quantitatively expressed in percent of a visual analog scale (VAS) at 4 and 8 min after SPT. Participants reported perceived itch intensity by pressing one of four buttons in an MRI-compatible button box (Current Design^TM^). Participants were familiarized with the rating procedure, which was a different reporting method than a previous study, with exercises before the fMRI session. This study did not record continuous ratings to minimize other effects.

### Skin Blood Perfusion Using Laser Doppler Perfusion Imaging

A PIM3 system (Perimed AB, Järfälla, Sweden) was used to measure skin blood perfusion in this study. The area of interest was 4.5 cm × 4.5 cm at the stimulus site on the non-dominant foot. The distance between the detector and the tissue was fixed to 45 cm with normal resolution. To minimize movement, the foot was fixed with a kapok-filled vacuum cushion. Skin blood perfusion was measured at baseline for 10 min after intervention (A1 and A2 phase), and 20 min post prick testing (H1, H2, H3, and H4 phase) in *Study 1*.

### Heart Rate Variability Using MP150

For electrocardiographic recordings, three electrodes were attached to participants’ chests. The sampling rate of the electrocardiogram (ECG) was 1000 samples/s and signals were amplified with a BIOPAC (Biopac Systems, Inc., Goleta, CA, United States) ECG100C amplifier. HRV data were calculated from a series of 5-min epochs of ECG signals. Spectral HRV components were evaluated and obtained as absolute values of power (ms^2^) based on frequency to one of three bands: very low frequency component (0.00–0.04 Hz), low frequency (LF) component (0.04–0.15 Hz), and high frequency (HF) component (0.15–0.40 Hz). HF and LF components of HRV were conventionally observed in normalized units. HF norm is an indicator of parasympathetic tone and LF norm is a measure of sympathetic regulation (Malliani et al., 1991; Camm et al., 1996). LF/HF ratio, an estimate of the balance between sympathetic and parasympathetic activities, was calculated from the absolute power of both frequency components.

### Blinding

At the end of the study, participants were asked if they believed they received “acupuncture treatment” or “placebo treatment.” Bang’s blinding index, ranging from −1 to 1, was calculated: 0 was random guessing (50% correct and 50% incorrect); 1 was complete unblinding (all responses correct); and −1 was all responses incorrect (Bang et al., 2010).

### Statistical Analysis

Statistical analyses were carried out using SPSS version 22.0 software (IBM SPSS Statistics, IBM Corporation, Armonk, NY, United States). Unless otherwise stated, data are mean values (95% confidence intervals). *P*-values <0.05 were considered statistically significant.

Data on itch intensity, skin blood perfusion, and HRV parameters were divided into 5-min intervals for ease of data management and analysis and changes were calculated using the formula: (change from baseline)/baseline X 100. Measurement times were divided into seven phases: (1) baseline, (2) A1 (0–5 min after acupuncture), (3) A2 (5–10 min after acupuncture), (4) H1 (0–5 min after histamine stimulus), (5) H2 (5–10 min after histamine stimulus), (6) H3 (10–15 min after histamine stimulus), (7) H4 (15–20 min after histamine stimulus). For responder and non-responder analyses, entire samples were separated into responders (*N* = 10) and non-responders (*N* = 10) based on the mean of sum values from arm and foot itch intensity at H1 using peak value.

Analyses were performed after checking all parameters were normally distributed using the Shapiro–Wilk one-sample test. Itch intensities for entire measurements were analyzed by two-way analysis of variance (ANOVA). Effects of time and time × treatment interaction using data divided into 5-min intervals were analyzed by repeated measure ANOVA. If Mauchly’s test of sphericity was significant, Greenhouse–Geisser correction for degrees of freedom was used. Differences between acupuncture and placebo conditions were evaluated using a paired sample *t*-test or Wilcoxon signed rank test. Blinding integrity was analyzed using a chi-square test. Differences between responder and non-responder groups were evaluated using independent sample *t*-test or Mann–Whitney test. Correlation analysis used Pearson’s correlation for parametric analysis.

### fMRI Data Acquisition

Scanning used a 3-axis gradient head coil in a 3 Tesla Siemens MRI scanner with echo planar imaging. Structural scans were acquired using a magnetization prepared rapid gradient echo sequence with TR = 2000 ms, TE = 2.37 ms, flip angle 9°, field of view 240 mm, and slice thickness 1.0 mm. For resting state analysis, 37 slices were acquired with parameters: TR = 2000 ms, TE = 30 ms, flip angle 90°, field of view 240 mm, and slice thickness 4.0 mm, with in-plane spatial resolution at 3 mm × 3 mm × 3 mm.

### fMRI Data Preprocessing and Analysis

This study focused only on resting state scans (baseline) before acupuncture and induced 8-min resting state scans (itch).

Preprocessing of resting state images used SPM12 software^1^ implemented in a MATLAB suite (Mathworks, Inc., Natick, MA, United States). Preprocessing included slice time correction, head motion correction, coregistration to patients’ structural images, segmentation, normalization, linear detrending, and smoothing (FWHM = 8 mm).

Functional connectivity analysis used the CONN toolbox ^2^ (Whitfield-Gabrieli and Nieto-Castanon, 2012). Time courses from components associated with white matter and cerebrospinal fluid were regressed from whole-brain gray matter activity and 12 motion regressors (6 realignment parameters and first derivatives) were used to control for correlations during movement. Data were filtered between 0.008 and 0.09 Hz and global brain signal was not subtracted.

Functional connectivity analysis used a seed-to-voxel approach. The left putamen was used as a seed. The segmentation mask of the left putamen in the Harvard-Oxford atlas is implemented in the CONN toolbox by default, so connectivity of the putamen with the anatomically defined brain areas was examined. The putamen mask used in our study (center MNI coordinates of *X* = 26, *Y* = 3, *Z* = −1) includes the MNI coordinates (*X* = −32, *Y* = −14, *Z* = 6) where is reported to be significantly correlated with itch rating in a previous study (Napadow et al., 2014). In first-level analysis, we produced a correlation map for each subject by extracting blood-oxygen-level dependent time courses from the putamen seed. Connectivity values between the putamen seed and other brain areas for the baseline and itch sessions (baseline-acupuncture and itch-acupuncture) were extracted for each participant. Furthermore, we performed Pearson correlation to explore the relationship between connectivity values of the putamen to VAS at 8 min, to determine if behavioral measures correlated with connectivity in the acupuncture conditions. Second-level connectivity analysis was performed with a between group factor (responder minus non-responder) and a within-subject factor treatment (itch minus baseline) to identify effects compared to baseline by two-way ANOVA. For all analyses, a threshold of *p* < 0.05, FDR corrected, was used based on the intensity threshold *p* < 0.001.

## Results

This study was conducted in two separate steps: *Study 1* was followed by *Study 2*. *Study 1* was 20 healthy participants (10 women, 10 men), mean age 22.5 (95% CI, 21.8–23.2) years, mean height 167.4 cm (95% CI, 163.5–171.3), mean weight 60.8 kg (55.8–65.9), and mean BMI 21.6 (95% CI, 20.3–22.9). From the 20 participants in *Study 1*, 15 (6 women, 9 men) who met the schedule for fMRI scans and retested for MRI compatibility, were mean age 22.8 (95% CI, 22.0–23.5) years, mean height 170.1 cm (95% CI, 166.0–174.1), mean weight 64.1 kg (59.0–69.2), and mean BMI 22.1 (95% CI, 20.7–23.5) and included in *Study 2*. *Study 1* and *Study 2* courses are in Figure 2. Participants had more than one experience with acupuncture and no present history of asthma or AD.

### Acupuncture vs. Placebo: Antipruritic Effects of Acupuncture

#### Changes in Itch Intensity

All of the 20 participants reported itch without pain 40 s after SPT. Itch intensities for the entire measurement were significantly lower in the acupuncture condition than the placebo condition for the arm (*p* < 0.001) and foot (*p* < 0.001) by two-way ANOVA (Figure 3B,C). Repeated-measure ANOVA showed significant effects on mean itch intensity for time (Greenhouse–Geisser corrected: *F* = 70.536, *p* < 0.001) and time × treatment interaction (Greenhouse–Geisser corrected: *F* = 7.486, *p* = 0.002) on the arm. Repeated-measure ANOVA showed significant effects on mean itch intensity for time (Greenhouse–Geisser corrected: *F* = 69.654, *p* < 0.001) and no significant effects on mean itch time × treatment interaction (Greenhouse–Geisser corrected: *F* = 2.150, *p* = 0.120) on the foot. At H1 and H2 phases, itch intensities were lower in the acupuncture condition (2.1 [95% CI, 1.4–2.7]; 1.3 [95% CI, 0.8–1.8]) compared to the placebo condition (3.8 [95% CI, 2.9–4.6]; 2.5 [95% CI, 1.5–3.5]) (*p* < 0.001 and *p* = 0.015, respectively) on the arm (Figure 3D). At H1, H2, and H3 phase, itch intensities were lower in the acupuncture condition (2.5 [95% CI, 1.7–3.4]; 1.6 [95% CI, 1.0–2.3]; 0.8 [95% CI, 0.4–1.2]) compared to the placebo condition (3.4 [95% CI, 2.6–4.1]; 2.6 [95% CI, 1.9–3.4]; 1.7 [95% CI, 1.0–2.3]) (*p* = 0.001, *p* = 0.001, and *p* = 0.002, respectively) on the foot (Figure 3E).

Time above scratch threshold was significantly lower in the acupuncture condition (21.7 [95% CI, 5.1–38.3]) than the placebo condition (71.4 [95% CI, 31.5–111.2], *p* = 0.002) on the arm. Time above scratch threshold was significantly lower in the acupuncture condition (29.3 [95% CI, 4.6–53.9]) than in the placebo condition (74.3 [95% CI, 33.3–115.3], *p* = 0.008) on the foot. The area under the rating curve above the scratch threshold for itch intensity was significantly lower in the acupuncture condition (94.2 [95% CI, −0.3 to 188.6]) than the placebo condition (568.3 [95% CI, 155.7–980.9], *p* = 0.001) on the arm (Figure 3F). The area under the rating curve above the scratch threshold for itch intensity was significantly lower in the acupuncture condition (178.1 [95% CI, −64.3 to 420.5]) than the placebo condition (473.7 [95% CI, 193.2–754.2], *p* = 0.008) on the foot (Figure 3G).

#### Changes in Skin Blood Perfusion

**FIGURE 4:**
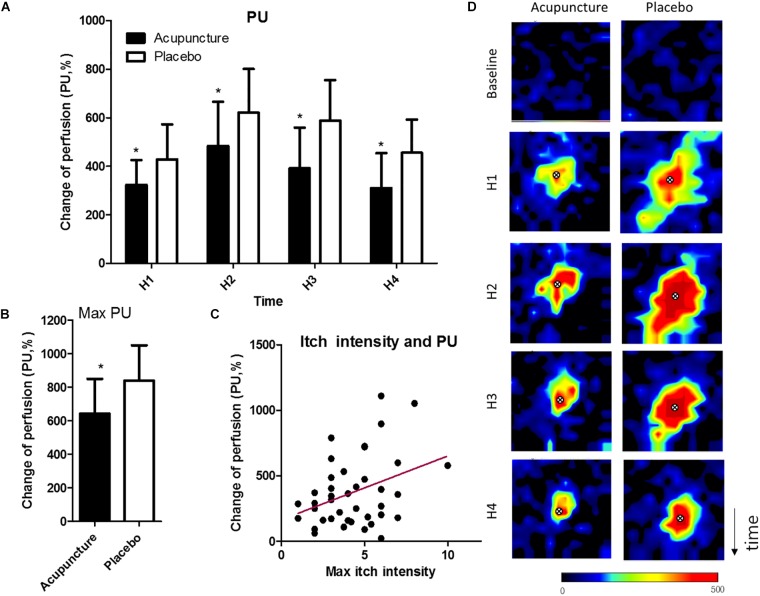
Changes in skin blood perfusion: **(A)** Mean of skin blood perfusion every 5 min after histamine stimulus, **(B)** Maximum skin blood perfusion during the study, **(C)** Correlation of maximum of itch intensity and skin blood perfusion, **(D)** Representative images of skin blood perfusion of a participant. X marks indicate the location of histamine injection. Values are mean (95% CI). *P*-values are for paired sample *t*-tests for comparison of change in skin blood perfusion from baseline between the two conditions. ^∗^*p* < 0.05. Perfusion unit ranges from black (lowest value) to red (highest). Relative to histamine stimulus, H1 is 0–5 min, H2 is 5–10 min, H3 is 10–15 min, and H4 is 15–20 20 min. PU, perfusion unit.

Mean perfusion units in the acupuncture condition were 323.3 (95% CI, 221.1–425.5), 391.5 (95% CI, 223.8–559.2), and 310.2 (95% CI, 166.9–453.6), and in the placebo condition 427.9 (95% CI, 283.6–572.1), 587.1 (95% CI, 419.7–754.4), 455.2 (95% CI, 318.6–591.8) (*p* = 0.049 at H1; *p* = 0.021 at H2; *p* = 0.011 at H3; *p* = 0.022 at H4) (Figure 4A). Maximum perfusion units were 642.8 (95% CI, 436.0–849.7) in the acupuncture condition and 840.6 (95% CI, 631.4–1050.0) in the placebo condition, with a significant difference (*p* = 0.036) (Figure 4B). Itch intensity was significantly correlated with skin blood perfusion (*r* = 0.36, *p* = 0.02) (Figure 4C). Representative images of skin blood perfusion from a participant are in Figure 4D.

#### Changes in Heart Rate Variability

Acupuncture compared to placebo showed no significant change on normalized high frequency (HF norm), normalized low frequency (LF norm), or LF/HF ratio. Participants in the acupuncture condition experienced increased HF norm and decreased LF norm and LF/HF ratio from baseline at H1, while participants in the placebo condition did not.

#### Assessment of Blinding

Comparisons of blinding rates showed no significant difference between conditions, as assessed by chi-square test with *p* = 0.068. Thus, participants could not predict if they received acupuncture or placebo. In the acupuncture condition, 75% of participants correctly guessed the treatment (Bang’s blinding index = 0.75 [95% CI, 0.52–0.98]). Placebo treatment had successful blinding (Bang’s blinding index = 0 [95% CI, −0.39 to 0.39]).

#### Seed-Based Functional Connectivity Analysis Using fMRI

Acupuncture stimulation (“itch” minus “baseline”) compared to placebo stimulation (“itch” minus “baseline”) revealed no significant change in functional connectivity of the putamen.

### Responder vs. Non-responder Analysis: Factors Influencing Acupuncture Response

For responder and non-responder analysis, the entire sample was separated into 10 responders and 10 non-responders in *Study 1*. They were based on a mean of the sum values of arm itch intensity and foot itch intensity at H1 in *Study 1* involving peak value in the acupuncture condition. Mean itch intensities at H1 phase in *Study 1* were 2.5 (95% CI, 1.9–3.1) in responders and 6.7 (95% CI, 4.9–8.5) in non-responders for sum values from arm and foot itch intensity in the acupuncture condition.

**FIGURE 5:**
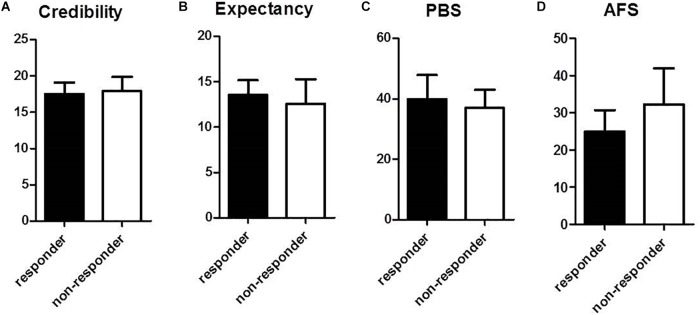
Comparison of psychological factors between responder and non-responder groups at baseline: **(A)** Questionnaire for acupuncture credibility, **(B)** Questionnaire for acupuncture expectancy, **(C)** Perception of bodily sensation, PBS, **(D)** Acupuncture Fear Scale. Values represent mean (95% CI).

Baseline data for psychological factors influencing response to acupuncture are in Figure 5. Questionnaire results for acupuncture credibility were not significantly different between responder and non-responder groups: values were 17.5 (95% CI, 15.9–19.1) in the responder group and 17.9 (95% CI, 15.9–19.9) in the non-responder group (Figure 5A). Acupuncture expectancy for the treatment indicated no significant difference between the responder (13.6 [95% CI, 11.9–15.2]) and non-responder groups (12.6 [95% CI, 9.9–15.3]) (Figure 5B). Perception of bodily sensation showed no significant difference between responder and non-responder groups: the value was 39.9 (95% CI, 31.9–47.9) in the responder group and 37.0 (95% CI, 31.0–43.0) in the non-responder group (Figure 5C). Fear of acupuncture was not significantly different between responder and non-responder groups. The scale was 25.0 (95% CI, 19.3–30.7) in the responder group and 32.3 (95% CI, 22.6–42.0) in the non-responder group (Figure 5D).

#### Changes in Itch Intensity

In *Study 1*, mean itch intensities at H1 phase were lower in responders (2.5 [95% CI, 1.9–3.1]) compared to non-responders (6.7 [95% CI, 4.9–8.5]) for sum values from arm and foot itch intensity (*p* < 0.001) in the acupuncture condition. In *Study 2*, mean itch intensities at 8 min were lower in responders (2.7 [95% CI, 0.7–4.6]) compared to non-responders (6.0 [95% CI, 4.0–8.1]) for sum values for arm and foot itch intensity (*p* = 0.021) in the acupuncture condition.

#### Changes in Heart Rate Variability

**FIGURE 6:**
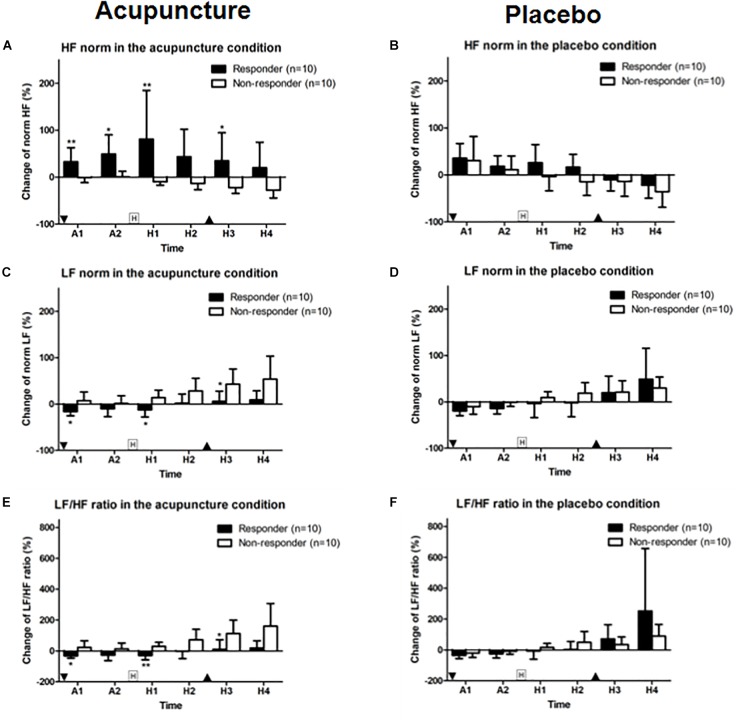
Change in HF and LF norm and LF/HF ratios: **(A,B)** HF norm, **(C,D)** LF norm, and **(E,F)** LF/HF ratio for acupuncture and placebo stimulation between responder and non-responder groups. Values are mean (95% CI). “Squared H” indicates time points for histamine stimulus. “Inverted triangle” indicates time point for insertion of acupuncture and “triangle,” removal of acupuncture. *P*-values are for independent sample *t*-tests comparing changes in heart rate variability from baseline between groups. ^∗^*p* < 0.05, ^∗∗^*p* < 0.01. A1 is 0–5 min and A2 is 5–10 min after intervention. Relative to histamine stimulus, H1 is 0–5 min, H2 is 5–10 min, H3 is 10–15 min, and H4 is 15–20 min.

HF norm in the responder group was 32.9 (95% CI, 3.0–62.9) and 49.5 (95% CI, 8.7–90.3). In the non-responder group, HF norm was −1.0 (95% CI, −11.7 to 9.8) and 0.8 (95% CI, −10.9 to 12.5) at A1 and A2 phase with a significant difference (*p* = 0.009 for A1 and *p* = 0.035 for A2). HF norm in the responder group was 81.2 (95% CI, −22.6 to 184.9) and 34.9 (95% CI, −24.8 to 94.7). HF norm in the non-responder group was −9.9 (95% CI, −17.2 to 2.6) and −22.1 (95% CI, −34.7 to 9.5) after histamine stimulus (H1 and H3 phase) with a significant difference (*p* = 0.007 for H1 and *p* = 0.029 for H3) between responder and non-responders to acupuncture (Figure 6A). HF norm values were not significantly different between responders and non-responders for placebo (Figure 6B).

LF norm for responders to acupuncture was −16.18 (95% CI, −25.3 to 7.1) for A1 phase, −12.4 (95% CI, −27.7 to 2.9) for H1 and 5.8 (95% CI, −16.4 to 28.0) for H3. LF norm for non-responders was 7.1 (95% CI, −12.1 to 26.3) for A1, 13.8 (95% CI, −2.7 to 30.2) for H1 and 42.9 (95% CI, 10.1–75.7) for H3. Differences were significant (*p* = 0.028, *p* = 0.015, and *p* = 0.023, respectively) (Figure 6C). LF norm values were not significantly different between responders and non-responders in the placebo condition (Figure 6D).

The LF/HF ratios in responders to acupuncture were −31.8 (95% CI, −46.9 to 16.7) for A1 phase, −29.0 (95% CI, −58.1 to 0.0) for H1 and 11.2 (95% CI, −49.9 to 72.4) for H3. LF/HF ratios for non-responders to acupuncture were 22.8 (95% CI, −19.9 to 65.5) for A1, 29.3 (95% CI, 2.9–55.8) for H1, and 1127 (95% CI, 26.2–199.3) for H3. Differences were significant (*p* = 0.019, *p* = 0.003, and *p* = 0.019, respectively) (Figure 6E). HF norm values were not significantly different between responders and non-responders for placebo given (Figure 6F).

#### Seed-Based Functional Connectivity Analysis Using fMRI

**FIGURE 7:**
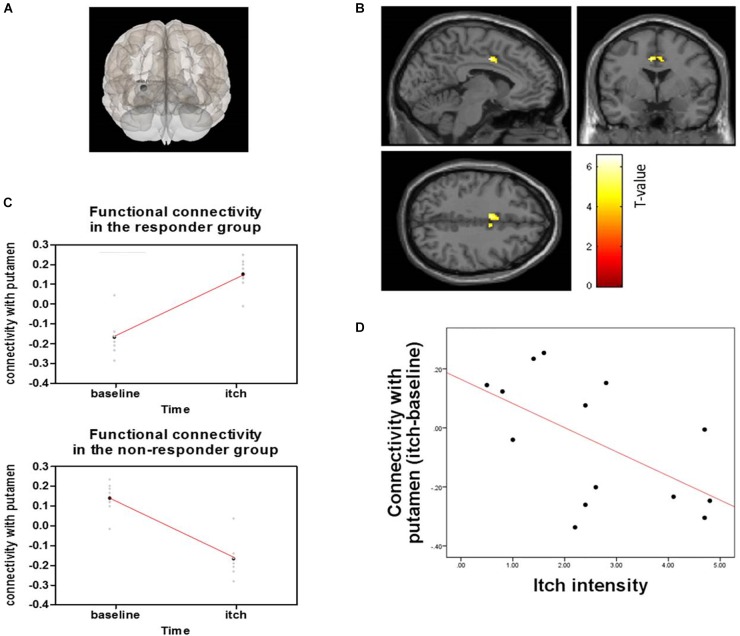
Functional connectivity of the putamen and pMCC and correlation with connectivity and itch intensity for acupuncture treatment between responder and non-responder groups: **(A)** Seed was putamen, **(B)** Seed-based functional connectivity from the putamen, showing a significant cluster in the midcingulate cortex (*X* = –6, *Y* = 0, *Z* = 40), **(C)** Change in functional connectivity from baseline resting state to histamine-induced itch state in responders (top) and non-responders (bottom), **(D)** Graph shows differences in connectivity from baseline to itch as a function of differences in perceived itch intensity from baseline to itch, as reported by participants for each scan session.

Responder (“itch” minus “baseline”) compared to non-responder showed significant changes in putamen-posterior part of the midcingulate cortex (pMCC) connectivity (MNI coordinates of the peak voxel: *X* = −6, *Y* = 0, *Z* = 40, cluster *p*-FDR = 0.043). More positive putamen-pMCC connectivity was observed in the responder group following acupuncture stimulation compared to the non-responder group. Connectivity correlated negatively with itch intensity (*r* = −0.573 *p* = 0.032) (Figure 7). Functional connectivity changed significantly from baseline to itch for several of seed regions including basal ganglia (peak *p*-unc <0.001) (Table 1).

**Table 1:** Brain regions showing functional connectivity of basal ganglia.

Seed region	Atlas label	Cluster (x, y, z)	Contrast	Size	Cluster *p*-FDR	Cluster *p*-unc
Putamen (l)	pMCC	(−6, 0, +40)	Positive	88	0.043	0.001
	aPaHC (l)	(−28, +10, +04)	Positive	92	0.036	0.001
Putamen (r)	aPaHC (l)	(+30, −06, −02)	Positive	65	0.040	0.004
Pallidum (l)	SubCalC	(−02, +26, −10)	Positive	84	0.049	0.003
	PaCiG (r)	(+06, +10, +46)	Positive	48	0.049	0.004
	FP (r)	(+42, +36, −16)	Negative	168	0.002	*p* < 0.001

*FDR, false discovery rate; unc, uncorrection; l, left; r, right; pMCC, posterior part of the midcingulate cortex; aPaHC, anterior parahippocampal gyrus; SubCalC, subcallosal cortex; PaCiG, paracingulate gyrus; FP, frontal pole.*

## Discussion

This study showed that experimentally induced itch and skin blood perfusion that were increased by histamine injection were significantly reduced after acupuncture compared to placebo. Because itch is not a local symptom but a systemic symptom in clinical situations, we induced itch on both the arm and the foot simultaneously. Acupuncture reduced itch in both areas, indicating the systemic antipruritic effects of acupuncture. This study also showed via responder and non-responder analysis that itch intensity reduction was associated with PNS activation and positive functional connectivity of the putamen-pMCC following acupuncture. Psychological factors such as credibility, expectation, and fear of acupuncture and perception of bodily sensation were not related to itch response to acupuncture. To our knowledge, this is the first study to investigate factors related to acupuncture by distinguishing differences between responders and non-responders to itch.

In previous studies, the effects of acupuncture stimulation on histamine-induced itch were observed in healthy participants: Belgrade et al. (1984) and Lundeberg et al. (1987) reported reduced itch intensity using electroacupuncture stimulation during intradermal histamine injection. Pfab et al. (2005) showed significant reduced histamine-induced itch and wheal formation after acupuncture-point treatment compared to placebo-point treatment and no treatment; Kesting et al. (2006) showed ipsilateral acupuncture treatment significantly reduces areas of alloknesis (a sensory phenomenon that appears in area surrounding an itching source) after histamine application compared with contralateral and no-acupuncture treatment. Pfab et al. (Pfab et al., 2010, 2012a) showed that patients had significantly reduced type I hypersensitivity itch, which is commonly seen in AD. Acupuncture could therefore be useful for managing itch, urticarial or eczema in patients with AD. Pfab et al. (2011) also showed that itch intensity and basophil activation decreased with acupuncture in patients with AD from IgE-mediated allergy. And as did in the previous study, our study chose a protocol of pretreatment acupuncture and skin prick test (SPT) for histamine, to investigate the preventive antipruritic effects of acupuncture. Because the itch VAS is a subjective outcome, we used the laser Doppler perfusion imaging detecting skin blood perfusion to measure itch objectively.

The ANS innervates almost every part of the body and interacts with the immune system. In particular, the parasympathetic division of the ANS facilitates the immune response (Bear et al., 2016). Recent studies elucidated the role of the ANS in the itch mechanism (Chida et al., 2007; Cicek et al., 2008). In healthy participants, histamine stimulus induces an increase in the SNS from baseline (Tran et al., 2010). Stander and Steinhoff (2002) suggested that ANS dysfunction may contribute to itch in patients with AD. Kirchner et al. (2002) showed that the vagus nerve controls histamine-induced itch. Several studies (Backer et al., 2008) explored the action of acupuncture on the ANS by analyzing changes in HRV. These studies suggest that acupuncture stimulates parasympathetic activity (Kitagawa et al., 2014). In our study, participants in the responder group had an activated PNS and deactivated SNS, but non-responder groups did not. These results imply that an activated PNS might be associated with the antipruritic effect of acupuncture.

The PNS was more highly activated during the first 5 min after acupuncture insertion (A1 phase) in the placebo condition than in the acupuncture condition. A possible explanation for this phenomenon of slightly higher activation after acupuncture stimulation could be a belief in an acupuncture effect, which was shown in another placebo study (Darragh et al., 2015). A study demonstrated that the needling-specific component (somatosensory needling stimulation) induces sympathetic activation, whereas needling non-specific components (needling credibility by ritual/contextual influence) result in increased parasympathetic activation after acupuncture needling (Lee et al., 2014). This result could also explain the more strongly activated PNS in the placebo condition. In this study, placebo treatment was successfully blinded. A Bang’s blinding index of 0 indicated that it might be a credible placebo (Bang et al., 2010). After histamine stimulus, acupuncture resulted in more activation of the PNS than placebo (H1 phase), showing that acupuncture increased parasympathetic activity; however, no significant change was observed in HF norm, LF norm, or LF/HF ratio between the acupuncture and placebo conditions. This result was partially because of high deviations in the HRV of participants, as examined by Kim and Woo (2011) in a normal Korean population. The impact of age, gender, and mean heart rate on short-term HRV measurement might have affected the results (Tsuji et al., 1996; Jensen-Urstad et al., 1997; Agelink et al., 2001; Kuch et al., 2001; Parati and Di Rienzo, 2003).

This study indicated that acupuncture had antipruritic effects via the positive connectivity of putamen and the pMCC. Itch is perceived by activation of inflammatory mediators via the spinothalamic tract to the brain (Davidson et al., 2007), influencing cortical and subcortical structures (Dhand and Aminoff, 2013). The striatum has a prominent function in striato-thalamo-cortical circuits (Koob and Volkow, 2010). The basal ganglia consists of the caudate nucleus, the putamen, the globus pallidus (consisting of an internal segment and an external segment), and the subthalamic nucleus (Bear et al., 2016). The putamen and caudate together are called the striatum, which is the target of cortical input to the basal ganglia. The globus pallidus is the source of output to the thalamus. Previous studies indicate that experimentally induced itch activates a brain network that includes a premotor and supplementary motor area, the thalamus, and the cingulate, insular, inferior parietal, and prefrontal cortices in healthy individuals (Pfab et al., 2012b). Electroacupuncture activates the putamen (Napadow et al., 2005), which is implicated in salience detection, particularly for stimuli such as pain (Downar et al., 2003) and itch. Recent fMRI study of patients with AD (Napadow et al., 2014) showed that acupuncture reduced itch and itch-evoked activation of parts of the putamen, insula, prefrontal, and premotor cortex (Napadow et al., 2014). Among these brain regions, greater reduction in putamen response was associated with greater decrease of itch intensity following acupuncture. The putamen is the region implicating the motivation and habitual behavior underlying the urge to scratch, and thus they suggested that the changes of putamen response are closely correlated with the antipruritic effects of acupuncture (Napadow et al., 2014). Based on this previous finding, we selected the putamen as seed for functional connectivity analysis using fMRI. The cingulate cortex consists of anterior cingulate cortex (ACC), midcingulate cortex (MCC), and posterior cingulate cortex (PCC). Among them, MCC or posterior part of the ACC was activated during itch induction or itch related behaviors (i.e., scratch), and the activation in this regions might be linked with cognition or evaluation of itch (Hsieh et al., 1994; Drzezga et al., 2001; Mochizuki et al., 2003, 2017; Herde et al., 2007; Papoiu et al., 2012). In a previous study, seed-based functional connectivity from the right anterior MCC showed a significant cluster in the right putamen in AD (Desbordes et al., 2015). In contrast, in this study, the significant connectivity of putamen and pMCC was found especially in responder to acupuncture during itch. The primary role of pMCC is reflexive orientation of the body in space to sensory stimuli including noxious ones (Vogt, 2016). Numerous neuroimaging studies have reported the activation of the pMCC during pain processing including the heat pain (Erpelding et al., 2012), short-duration and early noxious stimuli (Peyron et al., 2000; Bentley et al., 2003; Vogt et al., 2003; Frot et al., 2008). The MCC areas are thought to be numerous submodalities of sensory cortical processing (Vogt, 2014) and be involved in premotor planning and in affective-motivational processing in itch (Schneider et al., 2008). The motor-related regions such as the MCC along with the supplementary motor area and reward system including the putamen are associated with the reflexive process of scratching an itch (Mochizuki et al., 2015; Vogt, 2016). Thus, the antipruritic effect of acupuncture may be associated with the pMCC. Identifying negative correlations with clinical outcomes would allow us to understand how the positive connectivity of the putamen and pMCC lead to reduced itch.

Still, our results cannot exclude the possibility of the influence of individual difference of itch perception and responsiveness to histamine apart from the factors related with response to acupuncture. In this protocol of pretreatment acupuncture and SPT for histamine to investigate the preventive antipruritic effects of acupuncture, as the baseline itch sensation data before acupuncture are not exist, responders and non-responders were determined using absolute values of the itch sensation obtained in the acupuncture condition rather than the optimal way of using subtracted value between baseline and endpoint. And we decided not to use the subtracted value between placebo and acupuncture condition as we thought it was inappropriate for responder analysis of this study based on the clinical perspective. Though we investigated the antipruritic effects of acupuncture against experimentally induced itch in healthy volunteers, we purposed not to distinguish acupuncture-specific factors, but to explore factors contributing the clinical antipruritic effects of acupuncture and we tried to focus on the total effect of acupuncture not excluding placebo factors in responder analysis. However, this might have led to another possible conclusion that responders in this study may be simply less responsive to itch. There are several studies on the individual difference of itch sensitivity in patients with AD or psoriasis which conclude that patients with chronic itch reacted with a higher itch response to histamine, suggesting somatosensory stimuli are processed in line with that patients’ main symptom through generic sensitization processes (van Laarhoven et al., 2007, 2013). A seed-based analysis of a previous fMRI study (Desbordes et al., 2015) on evoked itch perception in chronic itch patients revealed decreased functional connectivity from baseline resting state to the evoked-itch state between several itch-related brain regions, particularly the insular and cingulate cortices and basal ganglia, where decreased connectivity was significantly correlated with increased levels of perceived itch. In contrast, evoked itch increased connectivity between key nodes of the frontoparietal control network (superior parietal lobule and dorsolateral prefrontal cortex), where higher increase in connectivity was correlated with a lesser increase in perceived itch, suggesting that greater interaction between nodes of this executive attention network serves to limit itch sensation via enhanced top-down regulation. Although these result cannot be directly applied to our study with healthy participants, individual difference of itch perception can naturally exist among healthy individuals. Further studies on brain regions or network in healthy participants that explain why some subjects perceive less itch are needed. Another fMRI study (Napadow et al., 2015) with AD patients demonstrated that expectations and other psychological factors play a role in modulating itch perception in chronic itch patients. In our study, psychological factors such as credibility, expectation, and fear of acupuncture and perception of bodily sensation were not related to itch response to acupuncture. The level of perceived bodily sensation varies from person to person and might lead to different responses of brain activation. In this study, perception of bodily sensation showed no significant difference between responder and non-responder groups: the value was 39.9 (95% CI, 31.9–47.9) in the responder group and 37.0 (95% CI, 31.0–43.0) in the non-responder group (Figure 5C).

In this study, acupuncture response to anti-pruritic effects might be associated with an anti-inflammatory effect mediated by an activated PNS and increased functional connectivity of putamen-pMCC. Acupuncture is thought to multidimensionally modulate anticipatory, somatosensory, and cognitive re-appraisal circuitries. Considering that the putamen and the MCC are related with somatosensory processing (Downar et al., 2003; Almeida et al., 2004; Vogt, 2014), the somatosensory aspect of acupuncture might be important for its antipruritic effects. However, while acupuncture may also have peripheral mechanisms as a counter stimulus (Ward, 1996), in this study, we induced itch on both the arm and the foot simultaneously and acupuncture reduced itch in both areas, suggesting that the systemic antipruritic effects of acupuncture are mediated by multiple central nervous pathways (Davidson and Giesler, 2010). Other studies indicated that antipruritic effects of acupuncture may modulate TRPV1 activation (McDonald et al., 2015, 2016) or endogenous opioid peptides in the central nervous system (Han, 2004; Wang et al., 2005; Dougherty et al., 2008; Guo et al., 2008; Zheng et al., 2008; Pfab et al., 2012a). Han et al. (2008) suggested that the antipruritic effects of acupuncture treatment are mediated by kappa-opioid receptor activation in a rat AD model. In addition to anti-inflammatory effects, the response of the sensory nerve could be a possible explanation for the antipruritic effects of acupuncture.

This study had several limitations that warrant consideration. First, we performed responder and non-responder analysis of itch intensity. Because we did not have a clinical standard due to the experimentally induced itch, entire participants were separated into responders and non-responders based on a mean of the sum values of arm and foot itch intensities. As mentioned above, responders and non-responders were determined using absolute values of the itch sensation obtained in the acupuncture condition, the possibility of the influence of individual difference of itch perception and responsiveness to histamine apart from the factors related with response to acupuncture still remains. Second, our model was not clinical itch symptoms, but experimentally induced itch. Third, this study also had a small sample size. By classifying into responder and non-responder participants, the sample numbers became even smaller considering that recommended sample size for fMRI studies is usually 16 or more. Thus, this study should be regarded as a pilot study requiring further studies with larger sample size and additional normal control group without any interventions to compensate the defect appeared in this pilot study and to induce definite conclusion. Next, according to the Bang’s blinding indexes for this study, the experimental condition was unblinded with 75% of correct guessing, while the control condition was completely blinded. Although this result can be interpreted as indicating that tactile stimulation acted as a credible placebo, the greater treatment effect in the experimental condition compared to control condition might have affected the study results (Park et al., 2008). Finally, no differences were seen in the analysis of the acupuncture and placebo conditions using HRV and fMRI data. As an explanation for the HRV results, acupuncture can be divided into needling-specific and non-specific components and it is equally applied to the placebo tactile stimulation (Langevin et al., 2011). Considering that the putamen and the pMCC are somatosensory processing regions related to afferent pain stimulation (Downar et al., 2003; Almeida et al., 2004; Vogt, 2014), the acupuncture and placebo condition might have had common brain responses.

## Conclusion

In conclusion, the results suggested that acupuncture treatment was useful against histamine-induced itch. Additionally, an activated PNS and the functional connectivity of putamen-pMCC could be considered factors related to the antipruritic response of acupuncture. Explaining clear mediators of the observed effects is still difficult. Thus, further work on the mechanisms involved in specific regulatory factors is needed. Further study to identify the most important brain region for the antipruritic effects of acupuncture with more participants is warranted. These factors might allow better prediction of the therapeutic effects of acupuncture.

## Data Availability

The raw data supporting the conclusions of this manuscript will be made available by the authors, without undue reservation, to any qualified researcher.

## Author Contributions

SM and K-WK involved in the manuscript preparation. SM and H-JP conceived and designed the experiments. W-MJ, M-JL, and Y-KK made substantial contributions to the study conception and performed the overall experiments. K-WK, YC, and HL provided the critiques and revised the article for important intellectual content. H-JP participated in the critical revision of the manuscript and had the final responsibility for the decision to submit for publication. All authors read and approved the final manuscript.

## Conflict of Interest Statement

The authors declare that the research was conducted in the absence of any commercial or financial relationships that could be construed as a potential conflict of interest. The handling Editor is currently organizing a Research Topic with one of the authors YC and confirms the absence of any other collaboration.

## Footnotes

1 http://www.fil.ion.ucl.ac.uk/spm

2 www.nitrc.org/projects/conn

## References

[B1] Agelink M. W. Malessa R. Baumann B. Majewski T. Akila F. Zeit T. (2001). Standardized tests of heart rate variability: normal ranges obtained from 309 healthy humans, and effects of age, gender, and heart rate. *Clin. Auton. Res.* 11 99–108. 10.1007/BF02322053 11570610

[B2] Almeida T. F. Roizenblatt S. Tufik S. (2004). Afferent pain pathways: a neuroanatomical review. *Brain Res.* 1000 40–56. 10.1016/j.brainres.2003.10.073 15053950

[B3] Andrew D. Craig A. D. (2001). Spinothalamic lamina I neurons selectively sensitive to histamine: a central neural pathway for itch. *Nat. Neurosci.* 4 72–77. 10.1038/82924 11135647

[B4] Arck P. Paus R. (2006). From the brain-skin connection: the neuroendocrine-immune misalliance of stress and itch. *Neuroimmunomodulation* 13 347–356. 10.1159/000104863 17709957

[B5] Backer M. Grossman P. Schneider J. Michalsen A. Knoblauch N. Tan L. (2008). Acupuncture in migraine: investigation of autonomic effects. *Clin. J. Pain* 24 106–115. 10.1097/AJP.0b013e318159f95e 18209515

[B6] Bang H. Flaherty S. P. Kolahi J. Park J. (2010). Blinding assessment in clinical trials: a review of statistical methods and a proposal of blinding assessment protocol. *Clin. Res. Regul. Aff.* 27 42–51. 10.3109/10601331003777444

[B7] Bear M. F. Connors B. W. Paradiso M. A. (eds) (2016). *Neuroscience: Exploring the Brain*. Philadelphia, PA: Wolters Kluwer.

[B8] Beissner F. Deichmann R. Henke C. Bar K. J. (2012). Acupuncture–deep pain with an autonomic dimension? *Neuroimage* 60 653–660. 10.1016/j.neuroimage.2011.12.045 22227140

[B9] Belgrade M. J. Solomon L. M. Lichter E. A. (1984). Effect of acupuncture on experimentally induced itch. *Acta Derm. Venereol.* 64 129–133. 6203300

[B10] Bender B. G. Ballard R. Canono B. Murphy J. R. Leung D. Y. (2008). Disease severity, scratching, and sleep quality in patients with atopic dermatitis. *J. Am. Acad Dermatol.* 58 415–420. 10.1016/j.jaad.2007.10.010 18280338

[B11] Bentley D. E. Derbyshire S. W. Youell P. D. Jones A. K. (2003). Caudal cingulate cortex involvement in pain processing: an inter-individual laser evoked potential source localisation study using realistic head models. *Pain* 102 265–271. 10.1016/S0304-3959(02)00405-0 12670668

[B12] Callahan S. W. Lio P. A. (2012). Current therapies and approaches to the treatment of chronic itch. *Dermatology* 17 29–40.

[B13] Camm A. Malik M. Bigger J. Breithardt G. Cerutti S. Cohen R. (1996). Heart rate variability: standards of measurement, physiological interpretation and clinical use. task force of the European society of cardiology and the North American society of pacing and electrophysiology. *Circulation* 93 1043–1065. 10.1161/01.CIR.93.5.1043 8598068

[B14] Chae Y. Park H. J. Hahm D. H. Yi S. H. Lee H. (2006). Individual differences of acupuncture analgesia in humans using cDNA microarray. *J. Physiol. Sci.* 56 425–431. 10.2170/physiolsci.RP010206 17083754

[B15] Charlesworth E. N. Beltrani V. S. (2002). Pruritic dermatoses: overview of etiology and therapy. *Am. J. Med.* 113(Suppl. 9A), 25S–33S. 10.1016/S0002-9343(02)01434-1 12517579

[B16] Chida Y. Steptoe A. Hirakawa N. Sudo N. Kubo C. (2007). The effects of psychological intervention on atopic dermatitis. A systematic review and meta-analysis. *Int. Arch. Allergy Immunol.* 144 1–9. 10.1159/000101940 17449959

[B17] Chung J. W. Yan V. C. Zhang H. (2014). Effect of acupuncture on heart rate variability: a systematic review. *Evid. Based Complement. Alternat. Med.* 2014:819871. 10.1155/2014/819871 24693326 PMC3944737

[B18] Cicek D. Kandi B. Berilgen M. S. Bulut S. Tekatas A. Dertlioglu S. B. (2008). Does autonomic dysfunction play a role in atopic dermatitis? *Br. J. Dermatol.* 159 834–838. 10.1111/j.1365-2133.2008.08756.x 18652587

[B19] Colombo B. Rocca M. A. Messina R. Guerrieri S. Filippi M. (2015). Resting-state fMRI functional connectivity: a new perspective to evaluate pain modulation in migraine? *Neurol. Sci.* 36(Suppl. 1), 41–45. 10.1007/s10072-015-2145-x 26017510

[B20] Darragh M. Vanderboor T. Booth R. J. Sollers J. J. III Consedine N. S. (2015). Placebo ’serotonin’ increases heart rate variability in recovery from psychosocial stress. *Physiol. Behav.* 145 45–49. 10.1016/j.physbeh.2015.03.043 25840004

[B21] Davidson S. Giesler G. J. (2010). The multiple pathways for itch and their interactions with pain. *Trends Neurosci.* 33 550–558. 10.1016/j.tins.2010.09.002 21056479 PMC2991051

[B22] Davidson S. Zhang X. Yoon C. H. Khasabov S. G. Simone D. A. Giesler G. J. Jr. (2007). The itch-producing agents histamine and cowhage activate separate populations of primate spinothalamic tract neurons. *J. Neurosci.* 27 10007–10014. 10.1523/JNEUROSCI.2862-07.2007 17855615 PMC3008349

[B23] Desbordes G. Li A. Loggia M. L. Kim J. Schalock P. C. Lerner E. (2015). Evoked itch perception is associated with changes in functional brain connectivity. *Neuroimage Clin.* 7 213–221. 10.1016/j.nicl.2014.12.002 25610783 PMC4300003

[B24] Dhand A. Aminoff M. J. (2013). The neurology of itch. *Brain* 137(Pt 2), 313–322. 10.1093/brain/awt158 23794605

[B25] Dhond R. P. Kettner N. Napadow V. (2007a). Do the neural correlates of acupuncture and placebo effects differ? *Pain* 128 8–12. 10.1016/j.pain.2007.01.001 17267130 PMC1913212

[B26] Dhond R. P. Kettner N. Napadow V. (2007b). Neuroimaging acupuncture effects in the human brain. *J. Altern. Complement. Med.* 13 603–616. 10.1089/acm.2007.7040 17718643

[B27] Dhond R. P. Yeh C. Park K. Kettner N. Napadow V. (2008). Acupuncture modulates resting state connectivity in default and sensorimotor brain networks. *Pain* 136 407–418. 10.1016/j.pain.2008.01.011 18337009 PMC2440647

[B28] Dougherty D. D. Kong J. Webb M. Bonab A. A. Fischman A. J. Gollub R. L. (2008). A combined [11C]diprenorphine PET study and fMRI study of acupuncture analgesia. *Behav. Brain Res.* 193 63–68. 10.1016/j.bbr.2008.04.020 18562019 PMC2538486

[B29] Downar J. Mikulis D. J. Davis K. D. (2003). Neural correlates of the prolonged salience of painful stimulation. *Neuroimage* 20 1540–1551. 10.1016/S1053-8119(03)00407-5 14642466

[B30] Drzezga A. Darsow U. Treede R. D. Siebner H. Frisch M. Munz F. (2001). Central activation by histamine-induced itch: analogies to pain processing: a correlational analysis of O-15 H2O positron emission tomography studies. *Pain* 92 295–305. 10.1016/S0304-3959(01)00271-8 11323151

[B31] Erpelding N. Moayedi M. Davis K. D. (2012). Cortical thickness correlates of pain and temperature sensitivity. *Pain* 153 1602–1609. 10.1016/j.pain.2012.03.012 22516588

[B32] Frot M. Mauguière F. Magnin M. Garcia-Larrea L. (2008). Parallel procesiing of nociceptive A-delta inputs in SII and midcingulate cortex in humans. *J. Neurosci.* 28 944–952. 10.1523/JNEUROSCI.2934-07.2008 18216202 PMC6670999

[B33] Guo Z. L. Moazzami A. R. Tjen A. L. S. Longhurst J. C. (2008). Responses of opioid and serotonin containing medullary raphe neurons to electroacupuncture. *Brain Res.* 1229 125–136. 10.1016/j.brainres.2008.07.020 18656464 PMC2579924

[B34] Han J. B. Kim C. W. Sun B. Kim S. K. Lee M. G. Park D. S. (2008). The antipruritic effect of acupuncture on serotonin-evoked itch in rats. *Acupunct. Electrother. Res.* 33 145–156. 10.3727/036012908803861168 19301625

[B35] Han J. S. (2004). Acupuncture and endorphins. *Neurosci. Lett.* 361 258–261. 10.1016/j.neulet.2003.12.019 15135942

[B36] Herde L. Forster C. Strupf M. Handwerker H. O. (2007). Itch induced by a novel method leads to limbic deactivations a functional MRI study. *J. Neurophysiol.* 98 2347–2356. 10.1152/jn.00475.2007 17715198

[B37] Hsieh J. C. Hägermark O. Ståhle-Bäckdahl M. Ericson K. Eriksson L. Stone-Elander S. (1994). Urge to scratch represented in the human cerebral cortex during itch. *J. Neurophysiol.* 72 3004–3008. 10.1152/jn.1994.72.6.3004 7897505

[B38] Huang H. Zhong Z. Chen J. Huang Y. Luo J. Wu J. (2015). Effect of acupuncture at HT7 on heart rate variability: an exploratory study. *Acupunct. Med.* 33 30–35. 10.1136/acupmed-2013-010441 25476448

[B39] Huang S.-T. Chen G.-Y. Lo H.-M. Lin J.-G. Lee Y.-S. Kuo C.-D. (2005). Increase in the vagal modulation by acupuncture at neiguan point in the healthy subjects. *Am. J. Chin. Med.* 33 157–164. 10.1142/S0192415X0500276X 15844844

[B40] Jensen-Urstad K. Storck N. Bouvier F. Ericson M. Lindblad L. E. Jensen-Urstad M. (1997). Heart rate variability in healthy subjects is related to age and gender. *Acta Physiol. Scand.* 160 235–241. 10.1046/j.1365-201X.1997.00142.x 9246386

[B41] Kawakita K. Shinbara H. Imai K. Fukuda F. Yano T. Kuriyama K. (2006). How do acupuncture and moxibustion act? - Focusing on the progress in Japanese acupuncture research. *J. Pharmacol. Sci.* 100 443–459. 10.1254/jphs.CRJ06004X 16799260

[B42] Kesting M. R. Thurmuller P. Holzle F. Wolff K. D. Holland-Letz T. Stucker M. (2006). Electrical ear acupuncture reduces histamine-induced itch (alloknesis). *Acta Derm. Venereol.* 86 399–403. 10.2340/00015555-0115 16955182

[B43] Kim G. M. Woo J. M. (2011). Determinants for heart rate variability in a normal Korean population. *J. Korean Med. Sci.* 26 1293–1298. 10.3346/jkms.2011.26.10.1293 22022180 PMC3192339

[B44] Kim J. H. Min B. I. Schmidt D. Lee H. J. Park D. S. (2000). The difference between electroacupuncture only and electroacupuncture with manipulation on analgesia in rats. *Neurosci. Lett.* 279 149–152. 10.1016/S0304-3940(99)00994-5 10688051

[B45] Kim S. K. Moon H. J. Park J. H. Lee G. Shin M. K. Hong M. C. (2007). The maintenance of individual differences in the sensitivity of acute and neuropathic pain behaviors to electroacupuncture in rats. *Brain Res. Bull.* 74 357–360. 10.1016/j.brainresbull.2007.07.006 17845910

[B46] Kirchner A. Stefan H. Schmelz M. Haslbeck K. M. Birklein F. (2002). Influence of vagus nerve stimulation on histamine-induced itching. *Neurology* 59 108–112. 10.1212/WNL.59.1.108 12105316

[B47] Kitagawa Y. Kimura K. Yoshida S. (2014). Spectral analysis of heart rate variability during trigger point acupuncture. *Acupunct. Med.* 32 273–278. 10.1136/acupmed-2013-010440 24610637

[B48] Koob G. F. Volkow N. D. (2010). Neurocircuitry of addiction. *Neuropsychopharmacology* 35 217–238. 10.1038/npp.2009.110 19710631 PMC2805560

[B49] Kuch B. Hense H. W. Sinnreich R. Kark J. D. von Eckardstein A. Sapoznikov D. (2001). Determinants of short-period heart rate variability in the general population. *Cardiology* 95 131–138. 10.1159/000047359 11474158

[B50] Langevin H. M. Wayne P. M. Macpherson H. Schnyer R. Milley R. M. Napadow V. (2011). Paradoxes in acupuncture research: strategies for moving forward. *Evid. Based Complement. Alternat. Med.* 2011:180805. 10.1155/2011/180805 20976074 PMC2957136

[B51] Lee J. Napadow V. Kim J. Lee S. Choi W. Kaptchuk T. J. (2014). Phantom acupuncture: dissociating somatosensory and cognitive/affective components of acupuncture stimulation with a novel form of placebo acupuncture. *PLoS One* 9:e104582. 10.1371/journal.pone.0104582 25101637 PMC4125217

[B52] Lee S. Lee M. S. Choi J. Y. Lee S. W. Jeong S. Y. Ernst E. (2010). Acupuncture and heart rate variability: a systematic review. *Auton. Neurosci.* 155 5–13. 10.1016/j.autneu.2010.02.003 20304708

[B53] Li Z. Wang C. Mak A. F. Chow D. H. (2005). Effects of acupuncture on heart rate variability in normal subjects under fatigue and non-fatigue state. *Eur. J. Appl. Physiol.* 94 633–640. 10.1007/s00421-005-1362-z 15906076

[B54] Lundeberg T. Bondesson L. Thomas M. (1987). Effect of acupuncture on experimentally induced itch. *Br. J. Dermatol.* 117 771–777. 10.1111/j.1365-2133.1987.tb07359.x 3426954

[B55] Malliani A. Pagani M. Lombardi F. Cerutti S. (1991). Cardiovascular neural regulation explored in the frequency domain. *Circulation* 84 482–492. 10.1161/01.CIR.84.2.482 1860193

[B56] McDonald J. L. Cripps A. W. Smith P. K. (2015). Mediators, receptors, and signalling pathways in the anti-inflammatory and antihyperalgesic effects of acupuncture. *Evid. Based Complement. Alternat. Med.* 2015:975632. 10.1155/2015/975632 26339274 PMC4539069

[B57] McDonald J. L. Smith P. K. Smith C. A. Changli Xue C. Golianu B. Cripps A. W. (2016). Effect of acupuncture on house dust mite specific IgE, substance P, and symptoms in persistent allergic rhinitis. *Ann. Allergy Asthma Immunol.* 116 497–505. 10.1016/j.anai.2016.04.002 27156748

[B58] Michikami D. Kamiya A. Kawada T. Inagaki M. Shishido T. Yamamoto K. (2006). Short-term electroacupuncture at Zusanli resets the arterial baroreflex neural arc toward lower sympathetic nerve activity. *Am. J. Physiol. Heart Circ. Physiol.* 291 H318–H326. 10.1152/ajpheart.00975.2005 16501021

[B59] Min S. Lee H. Kim S. Y. Park J. Y. Chae Y. Park H. J. (2015). Local changes in microcirculation and the analgesic effects of acupuncture: a laser Doppler perfusion imaging study. *J. Altern. Complement. Med.* 21 46–52. 10.1089/acm.2013.0442 25354241 PMC4296745

[B60] Mochizuki H. Papoiu A. D. P. Nattlemper L. A. Lin A. C. Kraft R. A. Coghill R. C. (2015). Scratching induces overactivity in motor-related regions and reward system in chronic itch patients. *J. Invest. Dermatol.* 135 2814–2823. 10.1038/jid.2015.223 26076316

[B61] Mochizuki H. Schut C. Nattkemper L. A. Yosipovitch G. (2017). Brain mechanism of itch in atopic dermatitis and its possible alteration through non-invasive treatments. *Allergol. Int.* 66 14–21. 10.1016/j.alit.2016.08.013 27688121

[B62] Mochizuki H. Tashiro M. Kano M. Sakurada Y. Itoh M. Yanai K. (2003). Imaging of central itch modulation in the human brain using positron emission tomography. *Pain* 105 339–346. 10.1016/S0304-3959(03)00249-5 14499452

[B63] Napadow V. Li A. Loggia M. L. Kim J. Mawla L. Desbordes G. (2015). The imagined itch: brain circuitry supporting nocebo-induced itch in atopic dermatitis patients. *Allergy* 70 1485–1492. 10.1111/all.12727 26280659 PMC4609272

[B64] Napadow V. Li A. Loggia M. L. Kim J. Schalock P. C. Lerner E. (2014). The brain circuitry mediating antipruritic effects of acupuncture. *Cereb. Cortex* 24 873–882. 10.1093/cercor/bhs363 23222890 PMC3948489

[B65] Napadow V. Makris N. Liu J. Kettner N. W. Kwong K. K. Hui K. K. (2005). Effects of electroacupuncture versus manual acupuncture on the human brain as measured by fMRI. *Hum. Brain Mapp.* 24 193–205. 10.1002/hbm.20081 15499576 PMC6871725

[B66] Noda Y. Izuno T. Tsuchiya Y. Hayasaka S. Matsumoto K. Murakami H. (2015). Acupuncture-induced changes of vagal function in patients with depression: a preliminary sham-controlled study with press needles. *Complement. Ther. Clin. Pract.* 21 193–200. 10.1016/j.ctcp.2015.07.002 26256139

[B67] Papoiu A. D. Coghill R. C. Kraft R. A. Wang H. Yosipovitch G. (2012). A tale of two itches. Common features and notable differences in brain activation evoked by cowhage and histamine induced itch. *Neuroimage* 59 3611–3623. 10.1016/j.neuroimage.2011.10.099 22100770 PMC3288667

[B68] Parati G. Di Rienzo M. (2003). Determinants of heart rate and heart rate variability. *J. Hypertens.* 21 477–480. 10.1097/01.hjh.0000052455.40108.db 12640235

[B69] Park H. J. Kim S. T. Yoon D. H. Jin S. H. Lee S. J. Lee H. J. (2005). The association between the DRD2 TaqI A polymorphism and smoking cessation in response to acupuncture in Koreans. *J. Altern. Complement. Med.* 11 401–405. 10.1089/acm.2005.11.401 15992222

[B70] Park J. Bang H. Canette I. (2008). Blinding in clinical trials, time to do it better. *Complement. Ther. Med.* 16 121–123. 10.1016/j.ctim.2008.05.001 18534323

[B71] Peyron R. Laurent B. García-Larrea L. (2000). Functional imaging of brain responses to pain. A review and meta-analysis. *Neurophysiol. Clin.* 30 263–288. 10.1016/S0987-7053(00)00227-6 11126640

[B72] Pfab F. Athanasiadis G. I. Huss-Marp J. Fuqin J. Heuser B. Cifuentes L. (2011). Effect of acupuncture on allergen-induced basophil activation in patients with atopic eczema:a pilot trial. *J. Altern. Complement. Med.* 17 309–314. 10.1089/acm.2009.0684 21443446

[B73] Pfab F. Hammes M. Backer M. Huss-Marp J. Athanasiadis G. I. Tolle T. R. (2005). Preventive effect of acupuncture on histamine-induced itch: a blinded, randomized, placebo-controlled, crossover trial. *J. Allergy Clin. Immunol.* 116 1386–1388. 10.1016/j.jaci.2005.08.055 16337477

[B74] Pfab F. Huss-Marp J. Gatti A. Fuqin J. Athanasiadis G. I. Irnich D. (2010). Influence of acupuncture on type I hypersensitivity itch and the wheal and flare response in adults with atopic eczema - a blinded, randomized, placebo-controlled, crossover trial. *Allergy* 65 903–910. 10.1111/j.1398-9995.2009.02284.x 20002660

[B75] Pfab F. Kirchner M. T. Huss-Marp J. Schuster T. Schalock P. C. Fuqin J. (2012a). Acupuncture compared with oral antihistamine for type I hypersensitivity itch and skin response in adults with atopic dermatitis: a patient- and examiner-blinded, randomized, placebo-controlled, crossover trial. *Allergy* 67 566–573. 10.1111/j.1398-9995.2012.02789.x 22313287 PMC3303983

[B76] Pfab F. Valet M. Napadow V. Tolle T. R. Behrendt H. Ring J. (2012b). Itch and the brain. *Chem. Immunol. Allergy* 98 253–265. 10.1159/000336529 22767068

[B77] Pfab F. Schalock P. C. Napadow V. Athanasiadis G. I. Yosipovitch G. Ring J. (2013). Complementary integrative approach for treating pruritus. *Dermatol. Ther.* 26 149–156. 10.1111/dth.12031 23551371

[B78] Schneider G. Ständer S. Burgmer M. Driesch G. Heuft G. Weckesser M. (2008). Significan differences in central imaging of histamine-induced itch between atopic dermatitis and healthy subjects. *Eur. J. Pain* 12 834–841. 10.1016/j.ejpain.2007.12.003 18203636

[B79] Scholz J. Woolf C. J. (2002). Can we conquer pain? *Nat. Neurosci.* 5(Suppl.), 1062–1067. 10.1038/nn942 12403987

[B80] Schork N. J. (2015). Personalized medicine: time for one-person trials. *Nature* 520 609–611. 10.1038/520609a 25925459

[B81] Stander S. Steinhoff M. (2002). Pathophysiology of pruritus in atopic dermatitis: an overview. *Exp. Dermatol.* 11 12–24. 10.1034/j.1600-0625.2002.110102.x 11952824

[B82] Takakura N. Ogawa H. Iijima S. Nishimura K. Kanamaru A. Sibuya M. (1995). Effect of acupuncture at the Hoku point on vibration-induced finger flexion reflex in man: comparison between press needle technique, electroacupuncture, and in-situ technique. *Am. J. Chin. Med.* 23 313–318. 10.1142/S0192415X95000377 8571928

[B83] Tran B. W. Papoiu A. D. Russoniello C. V. Wang H. Patel T. S. Chan Y. H. (2010). Effect of itch, scratching and mental stress on autonomic nervous system function in atopic dermatitis. *Acta Derm. Venereol.* 90 354–361. 10.2340/00015555-0890 20574599

[B84] Tsuji H. Venditti F. J. Jr. Manders E. S. Evans J. C. Larson M. G. Feldman C. L. (1996). Determinants of heart rate variability. *J. Am. Coll. Cardiol.* 28 1539–1546. 10.1016/S0735-1097(96)00342-7 8917269

[B85] van Laarhoven A. I. Kraaimaat F. W. Wilder-Smith O. H. van de Kerkhof P. C. Cats H. van Riel P. L. (2007). Generalized and symptom-specific sensitization of chronic itch and pain. *J. Eur. Acad. Dermatol. Venereol.* 21 1187–1192. 10.1111/j.1468-3083.2007.02215.x 17894703

[B86] van Laarhoven A. I. Kraaimaat F. W. Wilder-Smith O. H. van Riel P. L. van de Kerkhof P. C. Evers A. W. (2013). Sensitivity to itch and pain in patients with psoriasis and rheumatoid arthritis. *Exp. Dermatol.* 22 530–534. 10.1111/exd.12189 23802713

[B87] Vogt B. A. (2014). Submodalities of emotion in the context of cingulate subregions. *Cortex* 59 197–202. 10.1016/j.cortex.2014.04.002 24933713

[B88] Vogt B. A. (2016). Midcingulate cortex: structure, connections, homologies, functions and diseases. *J. Chem. Neuroanat.* 74 28–46. 10.1016/j.jchemneu.2016.01.010 26993424

[B89] Vogt B. A. Berger G. R. Derbyshire S. W. (2003). Stuructural and functional dichotomy of human midcingulate cortes. *Eur. J. Neurosci.* 18 3134–3144. 10.1111/j.1460-9568.2003.03034.x 14656310 PMC2548277

[B90] Wang Y. Zhang Y. Wang W. Cao Y. Han J. S. (2005). Effects of synchronous or asynchronous electroacupuncture stimulation with low versus high frequency on spinal opioid release and tail flick nociception. *Exp. Neurol.* 192 156–162. 10.1016/j.expneurol.2004.11.003 15698629

[B91] Ward A. A. (1996). Spontaneous electrical activity at combined acupuncture and myofascial trigger point sites. *Acupunct. Med.* 14 75–79. 10.1136/aim.14.2.75

[B92] Whitfield-Gabrieli S. Nieto-Castanon A. (2012). *Conn*: a functional connectivity toolbox for correlated and anticorrelated brain networks. *Brain Connect.* 2 125–141. 10.1089/brain.2012.0073 22642651

[B93] Woodcock J. Witter J. Dionne R. A. (2007). Stimulating the development of mechanism-based, individualized pain therapies. *Nat. Rev. Drug Discov.* 6 703–710. 10.1038/nrd2335 17762885

[B94] Yu C. Zhang P. Lv Z. T. Li J. J. Li H. P. Wu C. H. (2015). Efficacy of acupuncture in itch: a systematic review and meta-analysis of clinical randomized controlled trials. *Evid. Based Complement. Alternat. Med.* 2015:208690. 10.1155/2015/208690 26064156 PMC4430643

[B95] Zheng Z. Guo R. J. Helme R. D. Muir A. Da Costa C. Xue C. C. (2008). The effect of electroacupuncture on opioid-like medication consumption by chronic pain patients: a pilot randomized controlled clinical trial. *Eur. J. Pain* 12 671–676. 10.1016/j.ejpain.2007.10.003 18035566

