# Supplementary material for: Acupuncture for Histamine-Induced Itch: Association With Increased Parasympathetic Tone and Connectivity of Putamen-Midcingulate Cortex
Source: Front Neurosci. 2019 Mar 12;13:215. doi: 10.3389/fnins.2019.00215 (PMC6423085; doi:10.3389/fnins.2019.00215)
Supplement: Supplementary file 1 [file Table_1.DOCX]

**Appendix 1. Acupuncture treatment details as recommended by STRICTA**

|  | Item | Details |
| --- | --- | --- |
| 1. Acupuncture rationale | (a) Style of acupuncture | Traditional Korean medical theory |
|  | (b) Reasoning for treatment provided, based on historical context, literature sources, and consensus methods, with references where appropriate | - Textbook on acupuncture and moxibustion [1]  - Related articles and references [2-10]  LI11 and SP10 are used frequently to treat itch in clinical and experimental studies [3-6]. To induce activation of the PNS, HT7 [7] and PC6 [8,9] were chosen. ST36 is the most frequently used acupoint for immune regulation and anti-inflammatory effects, and reducing SNS [10].  - Consensus of acupuncture and dermatology experts at several offline meetings |
|  | (c) Extent to which treatment was varied | For *Study 2*, due to fMRI system structural problems, LI11 was excluded. |
| 2. Details of needling | (a) Number of needle insertions per subject per session (mean and range where relevant) | 5 |
|  | (b) Names (or location if no standard name) of points used (uni/bilateral) | - LI11 (on the elbow at the midpoint of the end of the transverse cubital crease and lateral epicondyle of the humerus when the elbow is flexed)  - SP10 (on the medial aspect of the thigh, 6.6 cm above the medio-superior border of the patella, on the bulge of the medial portion of muscle quadriceps femoris when the knee is flexed)  - PC6 (on the palmar aspect of the forearm, 6.6 cm proximal to the middle point of the carpal fold, on the line connecting PC3 and PC7, between the tendons of muscle palmaris longus and muscle flexor carpi radialis)  - HT7 (on the wrist, at the ulnar end of the transverse crease, in the depression on the radial side of the tendon of the ulnar flexor muscle)  - ST36 (on the anterior aspect of the lower leg, 9.9 cm below ST35, middle finger from the anterior crest of the tibia) |
|  | (c) Depth of insertion, based on a specified unit of measurement, or on a particular tissue level | 6 to 20 mm  (LI11, 20 mm; SP10, 15 mm; PC6, 15 mm; HT7, 6 mm; ST36, 20 mm) |
|  | (d) Response sought (eg, de qi or muscle twitch response) | 'De qi' sensation and muscle contraction |
|  | (e) Needle stimulation (eg, manual, electrical) | Manipulation techniques were performed as tonifying and reducing, which involved bidirectional rotation (1 Hz, for 9 s repeated every 5 min) |
|  | (f) Needle retention time | - 20 min for *Study 1*  - 13 min for *Study 2* |
|  | (g) Needle type (diameter, length, and manufacturer or material) | - A sterilised stainless steel needle (0.20 mm diameter × 30 mm length, Haeng Lim Seo Won, Korea) for *Study 1*  - A nonmagnetic titanium sterile acupuncture needle (0.20 mm diameter, 40 mm length; DongBang Acupuncture Inc.; Boryeoung, Republic of Korea) for *Study 2* |
| 3. Treatment regimen | (a) Number of treatment sessions | Four: Two for *Study 1* and two for *Study 2* (The study design was a randomized, crossover trial in which each participant served as his or her own control. Participants received acupuncture and placebo stimulation in random order.) |
|  | (b) Frequency and duration of treatment sessions | Each session was separated by 7 days and occurred at the same time of the day. |
| 4. Other components of treatment | (a) Details of other interventions administered to the acupuncture group (eg, moxibustion, cupping, herbs, exercises, lifestyle advice) | - |
|  | (b) Setting and context of treatment, including instructions to practitioners, and information and explanations to patients | - The von Frey filament for placebo controls and acupuncture materials were prepared in advance and placed on a tray and covered so materials could not be seen by participants.  - Locations of acupoints and nonacupoints were blinded by self-produced blinding box. |
| 5. Practitioner background | Description of participating acupuncturists (qualification or professional affiliation, years in acupuncture practice, other relevant experience) | The same doctor of Korean medicine with a certified license by the Korean Ministry of Health and Welfare performed all acupuncture treatments. |
| 6. Control or comparator interventions | (a) Rationale for the control or comparator in the context of the research question, with sources that justify this choice | - Related articles and references [1,11]  - Consensus of acupuncture and dermatology experts at several offline meetings |
|  | (b) Precise description of the control or comparator. If sham acupuncture or any other type of acupuncture-like control is used, provide details as for Items 1 to 3 above. | - As a placebo control, tactile stimulation was performed by gentle tapping with a size 5.46 von Frey filament (Touch-Test Sensory Evaluator Instructions, North Coast Medical, Inc., CA) every 5 min over 20 min for *Study 1* and over 5 min for *Study 2*.  - Placebo acupoints were on the arm and leg using the same meridian system, but not acknowledged as acupoints by textbooks. (The nonacupoint for LI11 was on the lateral side of the arm at the midpoint of the line connecting LI11 and LI15; The nonacupoint for SP10 was on the medial aspect of the thigh at the midpoint of the line connecting SP10 and SP12; The nonacupoint for PC6 was on the palmar aspect of the forearm at the midpoint of the line connecting PC3 and PC7; The nonacupoint for HT7 was on the palmar aspect of the forearm at the midpoint of the line connecting HT3 and HT7; The nonacupoint for ST36 was on the anterior aspect of the lower leg in the center of the belly of the muscle tibialis anterior.  - Placebo procedures were carried out as acupuncture procedures. |

1. Committee on Compilation of Textbook in Society for Acupuncture & Moxibustion: **Acupuncture and Moxibustion Medicine**. Paju: Jipmoondang Publishing Company; 2012.
2. Heo, J., Ahn, S., Kim, N., Jeong, C., and Cha, W.: **Donguibogam**. Seoul: Ministry of Health & Welfare; 2012.
3. Pfab, F., Athanasiadis, G.I., Huss-Marp, J., Fuqin, J., Heuser, B., Cifuentes, L., et al. (2011). Effect of acupuncture on allergen-induced basophil activation in patients with atopic eczema:a pilot trial. *J Altern Complement Med* 17(4)**,** 309-314. doi: 10.1089/acm.2009.0684.
4. Pfab, F., Hammes, M., Backer, M., Huss-Marp, J., Athanasiadis, G.I., Tolle, T.R., et al. (2005). Preventive effect of acupuncture on histamine-induced itch: a blinded, randomized, placebo-controlled, crossover trial. *J Allergy Clin Immunol* 116(6)**,** 1386-1388. doi: S0091-6749(05)02051-8 [pii] 10.1016/j.jaci.2005.08.055.
5. Pfab, F., Huss-Marp, J., Gatti, A., Fuqin, J., Athanasiadis, G.I., Irnich, D., et al. (2010). Influence of acupuncture on type I hypersensitivity itch and the wheal and flare response in adults with atopic eczema - a blinded, randomized, placebo-controlled, crossover trial. *Allergy* 65(7)**,** 903-910. doi: ALL2284 [pii] 10.1111/j.1398-9995.2009.02284.x.
6. Pfab, F., Kirchner, M.T., Huss-Marp, J., Schuster, T., Schalock, P.C., Fuqin, J., et al. (2012a). Acupuncture compared with oral antihistamine for type I hypersensitivity itch and skin response in adults with atopic dermatitis: a patient- and examiner-blinded, randomized, placebo-controlled, crossover trial. *Allergy* 67(4)**,** 566-573. doi: 10.1111/j.1398-9995.2012.02789.x.
7. Huang, H., Zhong, Z., Chen, J., Huang, Y., Luo, J., Wu, J., et al. (2015). Effect of acupuncture at HT7 on heart rate variability: an exploratory study. *Acupunct Med* 33(1), 30-35. doi: acupmed-2013-010441 [pii] 10.1136/acupmed-2013-010441.
8. Huang, S.-T., Chen, G.-Y., Lo, H.-M., Lin, J.-G., Lee, Y.-S., and Kuo, C.-D. (2005). Increase in the vagal modulation by acupuncture at neiguan point in the healthy subjects. *Am J Chin Med* 33(01), 157-164.
9. Li, Z., Wang, C., Mak, A.F., and Chow, D.H. (2005). Effects of acupuncture on heart rate variability in normal subjects under fatigue and non-fatigue state. *Eur J Appl Physiol* 94(5-6), 633-640. doi: 10.1007/s00421-005-1362-z.
10. Michikami, D., Kamiya, A., Kawada, T., Inagaki, M., Shishido, T., Yamamoto, K., et al. (2006). Short-term electroacupuncture at Zusanli resets the arterial baroreflex neural arc toward lower sympathetic nerve activity. *Am J Physiol Heart Circ Physiol* 291(1), H318-326. doi: 00975.2005 [pii] 10.1152/ajpheart.00975.2005.
11. Lee, H., Bang, H., Kim, Y., Park, J., Lee, S., Lee, H., et al. (2011). Non-penetrating sham needle, is it an adequate sham control in acupuncture research? *Complement Ther Med* 19S, S41-S48. doi: 10.1016/j.ctim.2010.12.002.
